# Variable-temperature NMR spectroscopy for metabolite identification in biological materials†

**DOI:** 10.1039/d1ra05626c

**Published:** 2021-11-03

**Authors:** Ewa K. Nawrocka, Mateusz Urbańczyk, Kamil Koziński, Krzysztof Kazimierczuk

**Affiliations:** Centre of New Technologies, University of Warsaw ul. Banacha 2C 02-097 Warsaw Poland k.kazimierczuk@cent.uw.edu.pl; Faculty of Chemistry, University of Warsaw ul. Pasteura 1 02-093 Warsaw Poland; Institute of Physical Chemistry, Polish Academy of Sciences Kasprzaka 44/52 01-224 Warsaw Poland

## Abstract

Nuclear magnetic resonance is a “workhorse technique” used in metabolomics, complementary to mass spectrometry. Unfortunately, only the most basic NMR methods are sensitive enough to allow fast medical screening. The most common of them, a simple ^1^H NMR, suffers from low dispersion of resonance frequencies, which often hampers the identification of metabolites. In this article we show that ^1^H NMR spectra contain previously overlooked parameters potentially helpful in metabolite identification, namely the rates of temperature-induced changes of chemical shifts. We prove that they are reproducible between various metabolite mixtures and can be determined quickly when Radon transform is used to process the data.

## Abstract 2

Rates of temperature-induced changes of NMR chemical shifts are easy to measure and metabolite-specific spectral parameters.

## Graphical abstract

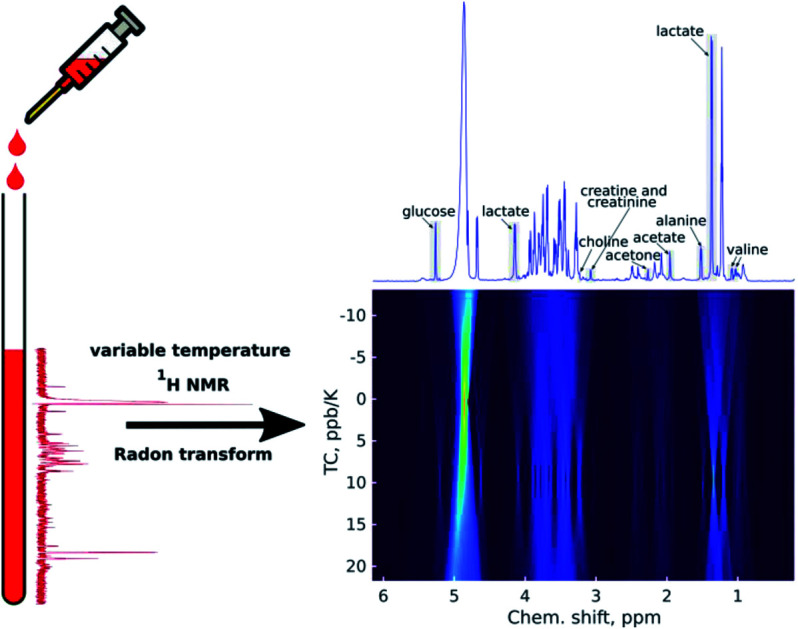

Nuclear magnetic resonance (NMR) spectroscopy plays an essential role in metabolomics research due to its unique characteristics, which include reproducibility, non-invasiveness and non-destructiveness.^1–3^ Many relevant experimental methods exist, including one-dimensional (1D) and two-dimensional (2D) NMR experiments that can be performed in a quantitative regime.^4^ 1D techniques can provide quick information about the presence of dozens of metabolites in a sample.^5–7^ However, the resolution of 1D spectra, and thus the specificity of the metabolite identification, is limited.^8^ 2D spectra overcome the problem of overlapping peaks, but at the cost of sensitivity and experimental time.^3,9^

Metabolomic research involves screening a multitude of samples^9^ whose stability is often relatively low. For this reason the measurements have to be rapid. This can be achieved, for example, by using new approaches based on non-uniform sampling (NUS)^10,11^ or ultra-fast spectroscopy.^12^

In this paper we propose a method for improving conventional NMR-based metabolite identification. The approach is fast, sensitive and based on an easily implementable concept of variable-temperature ^1^H NMR spectroscopy. The proposed method involves measuring not only ^1^H chemical shifts but also the rates of their temperature-induced changes.

These rates, referred to as temperature coefficients (TCs), are well known from structural studies of proteins, where they are used for characterising hydrogen bonds^13,14^ and confirming the presence of low-populated states.^15^ Yet, to the best of our knowledge, they have not previously been used for metabolite identification – although some values of metabolite temperature coefficients have been reported in the context of localised spectroscopy.^16^

To verify the feasibility of the new method, we formulated the following four research questions. First, are the changes of the metabolite chemical shifts linear in a reasonable temperature range? Second, are temperature coefficients characteristic for various metabolites? Third, are they reproducible between samples? And finally, what is the most effective way to measure them?

We studied samples of artificial and natural mixtures of metabolites. All samples were prepared in a sodium phosphate buffer (35 mM Na_2_HPO_4_; 6 : 4 (D_2_O : H_2_O), 3.05 mM NaN_3_; pH 7.4).^5,17^

We prepared three artificial mixtures of metabolites. The first was made from creatine (0.008 mM), creatinine (0.013 mM), l-alanine (0.054 mM), choline chloride (0.007 mM), l-valine (0.014 mM), glucose (0.559 mM), acetone (0.006 mM) and sodium acetate (0.022 mM). The second was made from l-leucine (0.003 mM), l-isoleucine (0.015 mM) and l-phenylalanine (0.008 mM). And the third was made from l-tyrosine (0.00325 mM), l-histidine (0.0151 mM) and (±)-sodium 3-hydroxybutyrate (S3HB, 0.0082 mM). In all three cases we used 3-(trimethylsilyl)propionic-2,2,3,3-*d*_4_ acid sodium salt (TMSP) as the chemical shift reference. We also added l(+)-lactic acid sodium salt (0.456 mM) to each of the mixtures as an internal reference for the temperature coefficient. We purchased the compounds from Sigma-Aldrich. The samples were placed in 5 mm NMR tubes.

In addition, we prepared a sample made of mouse serum. Mouse blood was collected by terminal procedure (intra-cardiac puncture). All of the experimental procedures were conducted in compliance with the current normative standards of the European Community (86/609/EEC) and the Polish Government (Dz.U. 2015 poz. 266). Animal usage was controlled by the institutional advisory board for animal welfare at the Centre of New Technologies. Serum was obtained by placing whole blood in an empty tube and allowing the blood to clot. Next, the sample was centrifuged and serum was transferred to a new tube. The sample was frozen in liquid nitrogen and stored in −80 °C until further analysis. Then, it was thawed at room temperature, shaken, and then filtered using a centrifugal filter with a modified nylon membrane (pore size 0.45 μm and working volume 500 μL) purchased from VWR. Next, 150 μL of mouse serum was dissolved into 150 μL of phosphate buffer with D_2_O (1 : 1), and 5 μL of TMSP (50 mM) was added. The solution was then transferred into a 3 mm NMR tube.

Finally, we purchased a certified human plasma NIST® SRM® 1950 sample (below referred to as NIST1950) from Sigma-Aldrich and prepared it according to the method described in Vignoli *et al.*^5^ and Simon-Manso *et al.*^17^ The purchased frozen vials were stored at −80 °C until needed. Prior to use, plasma ampules were thawed at room temperature, vortexed and filtered using a centrifugal filter with a modified nylon membrane (pore size 0.45 μm and working volume 500 μL) purchased from VWR. Next, 350 μL of the sample was added to 350 μL of a solution of a phosphate buffer with D_2_O (1 : 1). Finally, 600 μL of the solution was transferred to a 5 mm NMR tube and 10 μL of TMSP (50 mM) was added.

Measurements were performed using a 700 MHz Agilent DirectDrive2 spectrometer equipped with a room-temperature HCN probe. We used CPMG pulse sequence with 64K data points, an acquisition time of 2.94 s, and a length of CPMG pulse train of 0.5 s. The temperature was controlled in the following ranges: 10.73–33.82 °C for the NIST1950 sample, 24.20–39.59 °C for the first artificial mixture, 24.20–31.89 °C for the second and third artificial mixtures and 9.57–33.62 °C for the mouse serum. The temperature was calibrated using an ethylene glycol sample. The acquisitions of the spectra in a series were interleaved with temperature stabilisation delays of 10 minutes and automated gradient shimming, as well as adjusting the solvent presaturation frequency based on the assumption of linear shift of the water peak. We used a Python script based on the TReNDS library^18^ to implement these procedures.

For the NIST1950 plasma sample we also carried out a “fast” experiment to check if reliable temperature coefficients could be obtained in less time. To this end, we reduced the number of scans from 160 to 8, the temperature stabilisation period from 10 to 2 minutes, the interscan delay from 10 to 2 seconds, and did not carry out re-shimming between the spectra. In addition, we reduced the temperature range slightly (to 9.57–27.85 °C) so the experiment did not last longer than an hour.

We determined the limit of detection using the lactate peak (1.36 ppm at 25.16 °C): it was 6.51 μM for “fast” experiments and 35.57 μM for “slow” experiments.

We processed and analysed the 1D data sets using Mnova 12.0 software (Mestrelab Research, S. L., Spain) with zero-filling to 128K and an exponential weighting function (line broadening of 2 Hz). Phase and baseline corrections using the polynomial fit were performed automatically on each spectrum.

The rates of linear change of the chemical shifts were determined using a MATLAB (MathWorks, USA) script, fitting a line to the positions of peaks picked using Mnova 12.0 in each spectrum in a series. Below, we refer to this method as “traditional fitting”.

We also processed the data from the “fast” experiment on the NIST1950 sample using the Radon transform^19^ TIPSY script (*rt_2D*) from Bruker TopSpin 4.0.8 software (Bruker, USA), processing samples with zero-filling to 128K and an exponential weighting function (line broadening of 2 Hz). Zero and first-order phase correction using *pk* mode, and baseline correction using *quad* mode, were performed automatically on each spectrum using *proc1d y* script. We analysed the Radon spectra using the nmrDraw program from the nmrPipe package.^20^ Before Radon processing, however, we added artificial noise to the data. This provided a useful example of low-sensitivity measurement where “traditional fitting” was not possible as some of the peaks could not be found.

We began our analysis of the results by verifying the linearity of the chemical shift changes with temperature. The series of ^1^H NMR spectra as these shown in Fig. 1 were peak-picked and chemical shift plotted against temperature (see ESI†). For all samples, dependencies were highly linear in all the temperature ranges studied (*R*^2^ from 0.9892 to 0.99999).

**Fig. 1:**
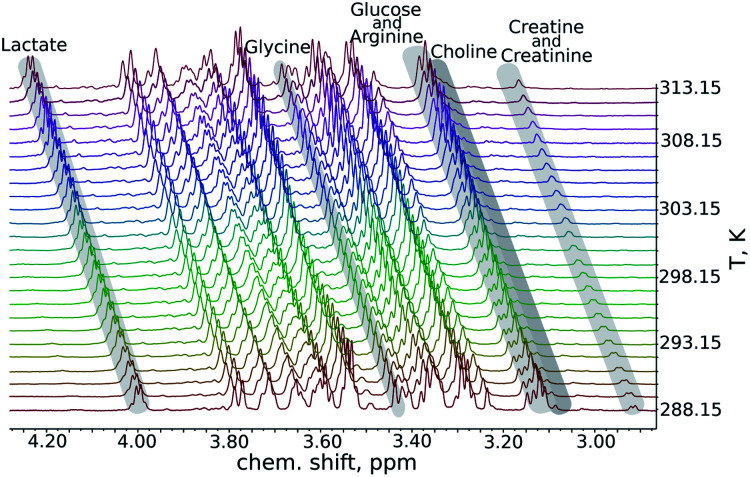
A fragment from a series of variable-temperature ^1^H NMR spectra. Peaks of key metabolites are marked. An important observation is that the rates of change of chemical shifts are peak-specific.

As can be seen in Fig. 2, the temperature coefficients of different groups of nuclei differ quite significantly. They can thus be used as an easy-to-measure parameter for peak assignment or metabolite identification. However, the necessary condition is that these characteristic differences should be reproducible between samples. We therefore compared the results of the artificial mixture and the natural mixtures (Fig. 2). As we observed a constant shift between the samples, which can be attributed to different changes of the deuterium lock frequency, we shifted all the temperature coefficients by a constant, treating the well-resolved, intense lactate peak (1.36 ppm at 25.16 °C for the artificial mixture of metabolites) as a reference, set to 10.75 ppb K^−1^. We did not use the water temperature coefficient as a reference as the water signal is sensitive to many factors other than temperature, such as pH, ionic strength and sample composition.^21–23^ For the same reason we did not subtract the water temperature coefficient from the data.

**Fig. 2:**
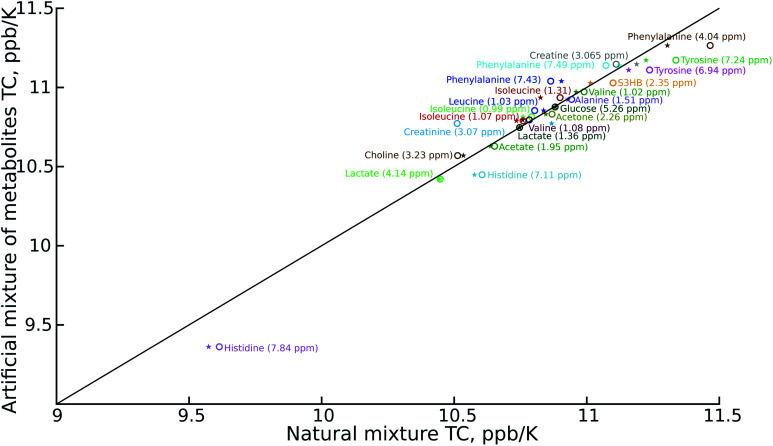
Comparison of temperature coefficients for: artificial mixtures of metabolites and certificated human plasma (NIST1950) (stars); and artificial mixtures of metabolites and mouse serum (circles). Different signals are shown in different colours. The temperature coefficient (TC) scale has been shifted to keep the lactate peak (dark green) at 10.75 ppb K^−1^. The chemical shift of the metabolites is taken from the spectra of the artificial mixtures of metabolites registered at 25.16 °C.

The reproducibility of the temperature coefficients for most of the metabolites is very good, except in the case of overlapping peaks. The peak overlap explains the distortion of creatine and creatinine temperature coefficients. This problem could be solved by 2D spectroscopy, for example by exploiting a temperature-swept 2D ^13^C HSQC, as described previously.^24,25^ On the other hand it is worth noting that even the distorted temperature coefficients of these two compounds are much more characteristic than chemical shifts alone, and thus distinguishing between creatine (high temperature coefficient) and creatinine (low temperature coefficient) might be a good example of using VT-NMR for metabolite identification.

The procedures that we used to measure the temperature coefficients were quite time-consuming compared to measuring a single ^1^H NMR spectrum: we applied 10-minutes temperature stabilisation delays before each spectrum in a series, as well as automated gradient re-shimming, giving a total experimental time of 21 hours. This clearly means that the method is unsuitable for large-scale metabolomic screening. However, shortening the temperature stabilisation delay to 2 minutes and doing without the re-shimming, plus cutting the number of scans from 160 to 8, allows us to reduce the experimental time to just one hour. As shown in Fig. 3, the results of this “fast” experiment are close to those of the “slow” experiment. In fact, only the determination of the choline peak position is significantly affected by the lack of shimming, which causes the neighbouring glucose peak to broaden.

**Fig. 3:**
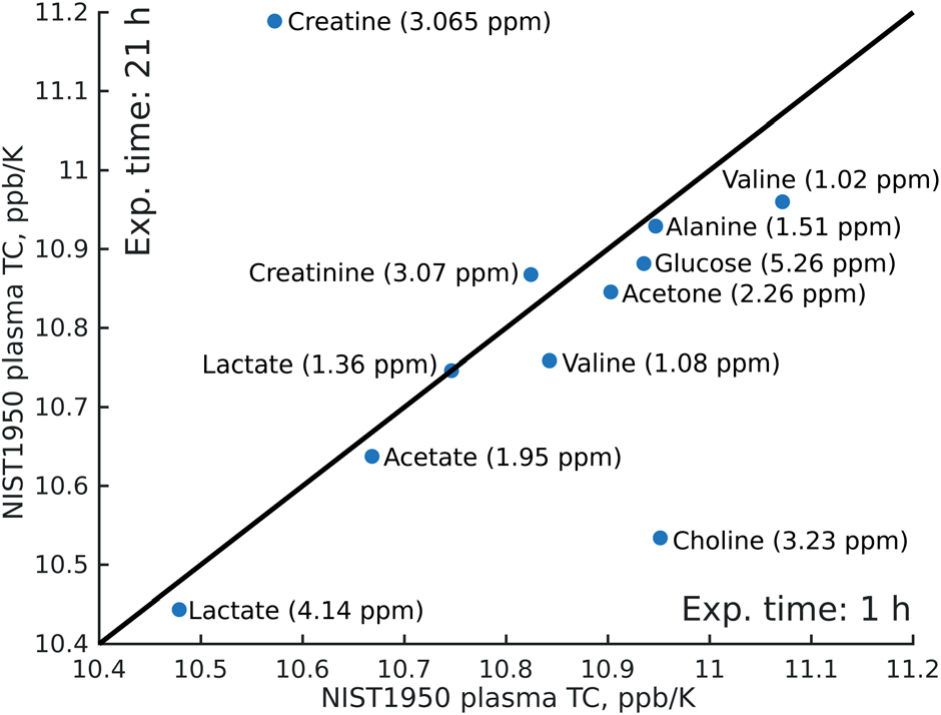
Comparison of temperature coefficients (TCs) obtained in an experiment lasting 1 hour and an experiment lasting 21 hours. Experiments were performed on a certified sample of human plasma (NIST1950). The chemical shift of the metabolites was taken from the spectrum of the artificial mixture of metabolites registered at 25.16 °C.

The reduction in sensitivity can be compensated for by using Radon transform processing.^19,25^ To demonstrate this, we added artificial noise to the data set acquired in 1 hour. As shown in Fig. 4c, some peaks in the “noisy” data (for example acetone or creatine/creatinine) are impossible to localise. However, they can easily be found in the 2D Radon spectrum (Fig. 4b). Moreover, the temperature coefficients obtained from this spectrum are in excellent agreement with those obtained by “traditional fitting” of the low-noise data (see Fig. 5). As explained in our previous papers, the 2D Radon peak has the same signal-to-noise ratio as a peak in a regular ^1^H NMR spectrum measured in almost the same total time as a serial experiment (neglecting temperature-stabilisation delays).^25^ So even for low-concentrated samples, temperature coefficients can be determined in a reasonable time by using Radon transform processing.

**Fig. 4:**
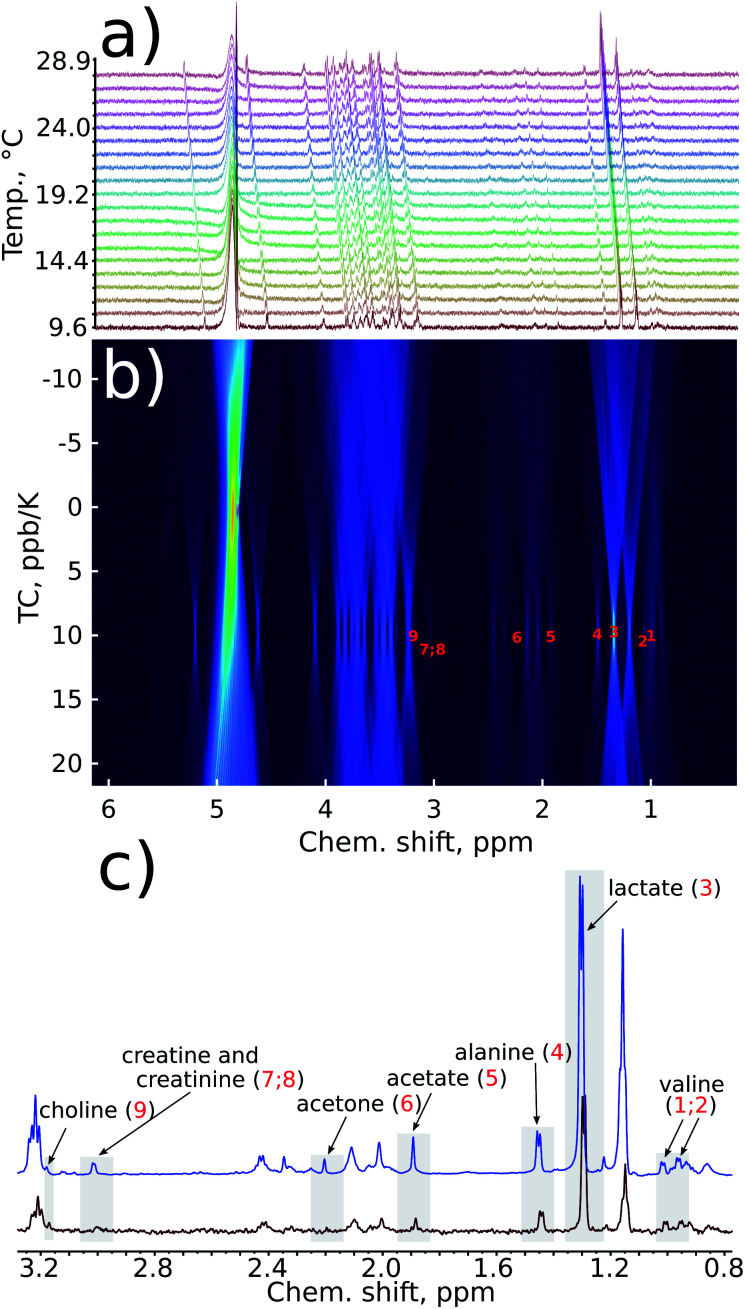
Sensitivity enhancement by a Radon transform: (a) series of 1D spectra of a certified sample of human plasma (NIST1950) with added artificial gaussian noise; (b) Radon transform of the data (TC stands for temperature coefficient); (c) comparison of the fragment of the first spectrum in a series with (red) and without (blue) artificial noise added. Some peaks are below the noise level in the first spectrum but visible in the Radon spectrum due to coherent addition of peaks.

**Fig. 5:**
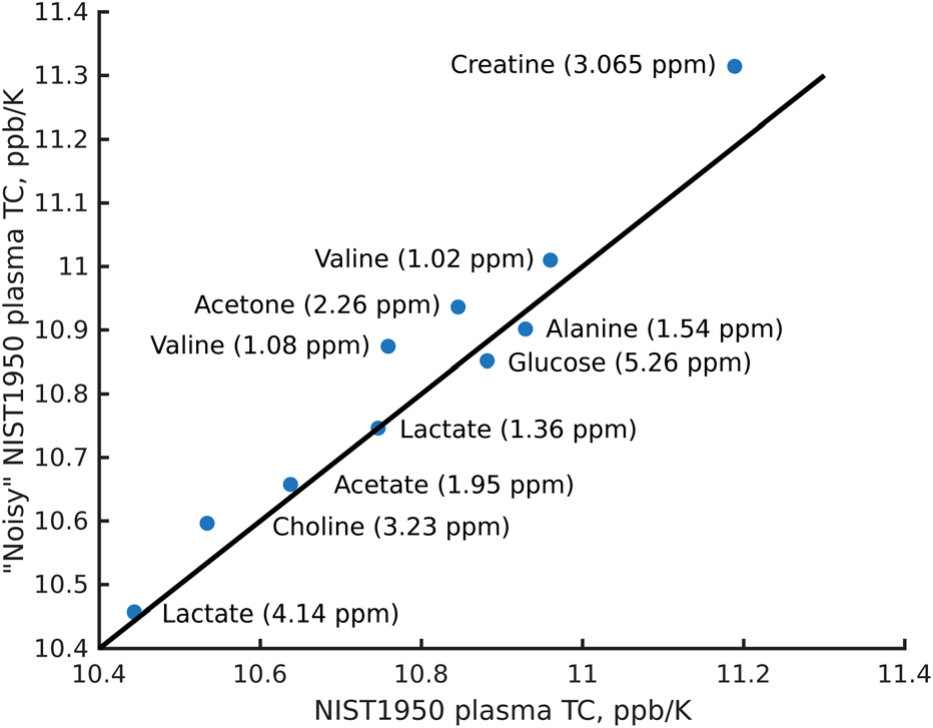
Comparison of the temperature coefficients (TCs) obtained from the Radon spectrum of certified human plasma (NIST1950) with noise added and an experiment obtained after “traditional fitting” of the NIST1950 data acquired in 21 hours. The chemical shifts of the metabolites were taken from the spectrum of the artificial mixture of metabolites registered at 25.16 °C.

To summarise, we have shown that the rate of change of a chemical shift with a temperature is a specific, easily measurable and reproducible parameter that can be used to identify metabolites in NMR spectra. This rate of change can be measured in roughly the same amount of time needed for standard multi-scan ^1^H NMR spectra. It therefore represents an additional method for dealing with difficult samples of metabolites. Moreover, it can be combined with other advanced methods of metabolomic NMR, such as 2D spectroscopy and similar.^26^ Recently, fast multidimensional experiments with a temperature-sweep parallel to the sampling of indirect time dimensions have been proposed.^24,25,27,28^ Versions of these temperature-sweep techniques could also be used in metabolomics.

Our study was limited to just a few common metabolites. However, the specificity and reproducibility of temperature coefficients that we observe suggests that it might be beneficial to collect and publish their values in publicly available databases, as is the case with chemical shifts.^29–31^ We further believe that the temperature coefficients of small molecules can be used in the analysis of other mixtures of natural origins, such as food, plant extracts and fuels. Although these temperature coefficients are difficult to interpret directly, as in the case of proteins (hydrogen bonding), their role as a “fingerprint” appears to be promising.

## Conflicts of interest

There are no conflicts to declare.

## Supplementary Material

RA-011-D1RA05626C-s001
