# Supplementary material for: Variable-temperature NMR spectroscopy for metabolite identification in biological materials
Source: RSC Adv. 2021 Nov 3;11(56):35321–5. doi: 10.1039/d1ra05626c (PMC9043013; doi:10.1039/d1ra05626c)
Supplement: RA-011-D1RA05626C-s001 [file RA-011-D1RA05626C-s001.pdf]

Electronic Supplementary Material (ESI) for RSC Advances.
This journal is © The Royal Society of Chemistry 2021

Supplementary information

for

Variable-temperature NMR spectroscopy for metabolite identification in biological materials

Ewa K. Nawrocka[a,b], Mateusz Urbańczyk[a,c], Kamil Koziński[a], and Krzysztof Kazimierczuk[a]

a) Centre of New Technologies, University of Warsaw, ul. Banacha 2C, 02-097 Warsaw, Poland
b) Faculty of Chemistry, University of Warsaw, Pasteura 1, 02-093 Warsaw, Poland
c) Institute of Physical Chemistry, Polish Academy of Sciences, Kasprzaka 44/52, 01-224 Warsaw, Poland

Chemical shift dependence, ppb, on temperature, °C, for metabolites from the first artificial mixture. Temperature coefficients (TCs) as a slope of the linear function. For all diagrams, the coefficient of determination R^2 is shown.

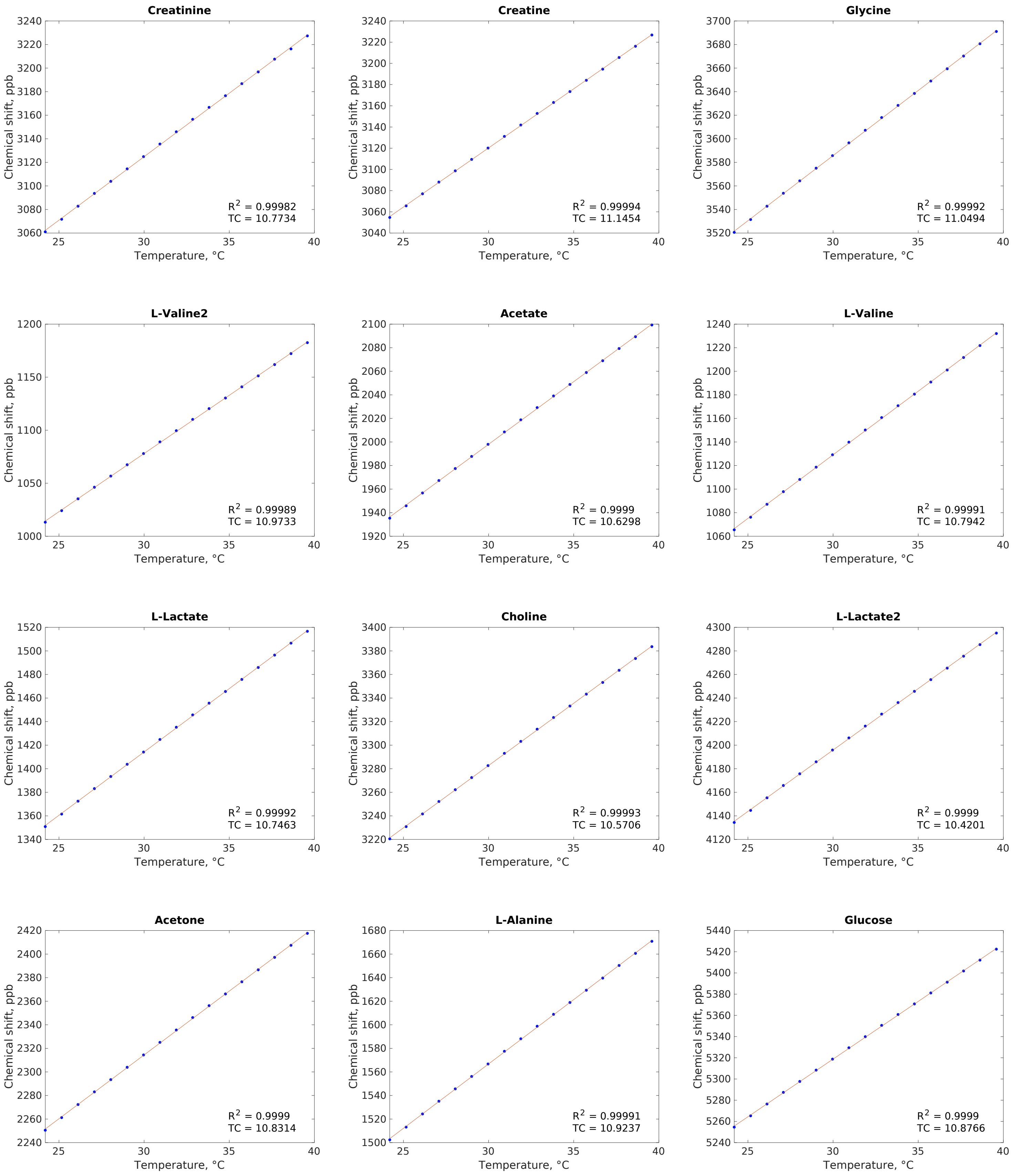
Creatinine
Chemical shift, ppb
Temperature, °C
R2 = 0.99982
TC = 10.7734
Creatine
R2 = 0.99994
TC = 11.1454
Glycine
R2 = 0.99992
TC = 11.0494
L-Valine2
R2 = 0.99989
TC = 10.9733
Acetate
R2 = 0.9999
TC = 10.6298
L-Valine
R2 = 0.99991
TC = 10.7942
L-Lactate
R2 = 0.99992
TC = 10.7463
Choline
R2 = 0.99993
TC = 10.5706
L-Lactate2
R2 = 0.9999
TC = 10.4201
Acetone
R2 = 0.9999
TC = 10.8314
L-Alanine
R2 = 0.99991
TC = 10.9237
Glucose
R2 = 0.9999
TC = 10.8766

Chemical shift dependence[1], ppb, on temperature, °C, for NIST1950 plasma sample (experiment lasting 21 h). Temperature coefficients (TCs) as a slope of the linear function. For all diagrams, the coefficient of determination R^2 is shown.

1 Raw data, before referencing to Lactate peak

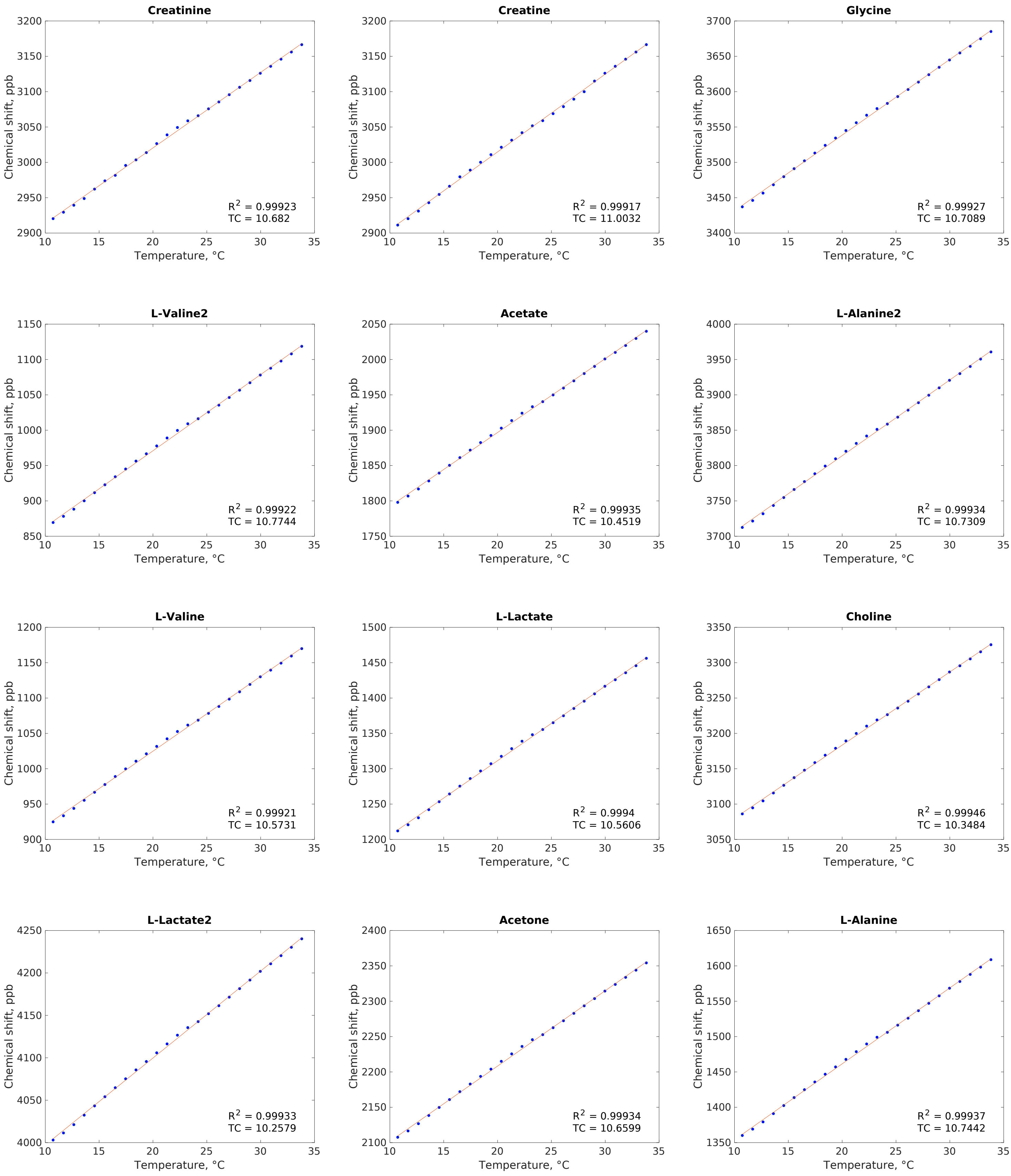

Creatinine
R^2 = 0.99923
TC = 10.682
Creatine
R^2 = 0.99917
TC = 11.0032
Glycine
R^2 = 0.99927
TC = 10.7089
L-Valine2
R^2 = 0.99922
TC = 10.7744
Acetate
R^2 = 0.99935
TC = 10.4519
L-Alanine2
R^2 = 0.99934
TC = 10.7309
L-Valine
R^2 = 0.99921
TC = 10.5731
L-Lactate
R^2 = 0.9994
TC = 10.5606
Choline
R^2 = 0.99946
TC = 10.3484
L-Lactate2
R^2 = 0.99933
TC = 10.2579
Acetone
R^2 = 0.99934
TC = 10.6599
L-Alanine
R^2 = 0.99937
TC = 10.7442
Chemical shift, ppb
Temperature, °C

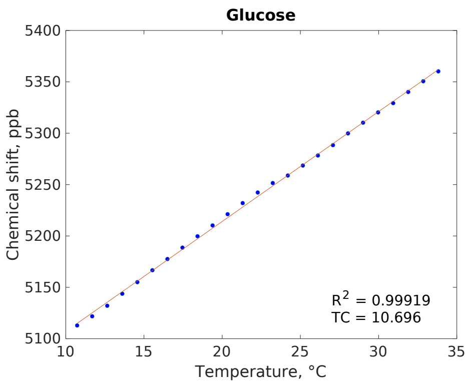
Glucose
Chemical shift, ppb
Temperature, °C
5400
5350
5300
5250
5200
5150
5100
10
15
20
25
30
35
R^2 = 0.99919
TC = 10.696

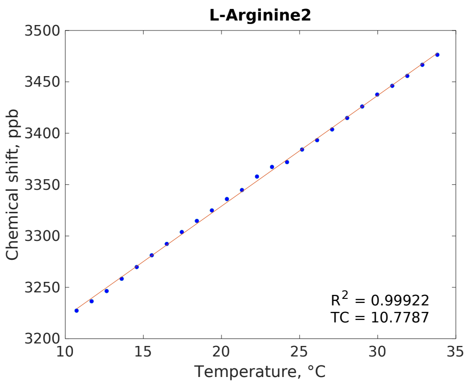
L-Arginine2
Chemical shift, ppb
Temperature, °C
3500
3450
3400
3350
3300
3250
3200
10
15
20
25
30
35
R^2 = 0.99922
TC = 10.7787

Chemical shift dependence[1], ppb, on temperature, °C, for mouse serum. Temperature coefficients (TCs) as a slope of the linear function. For all diagrams, the coefficient of determination R^2 is shown.

1 Raw data, before referencing to Lactate peak

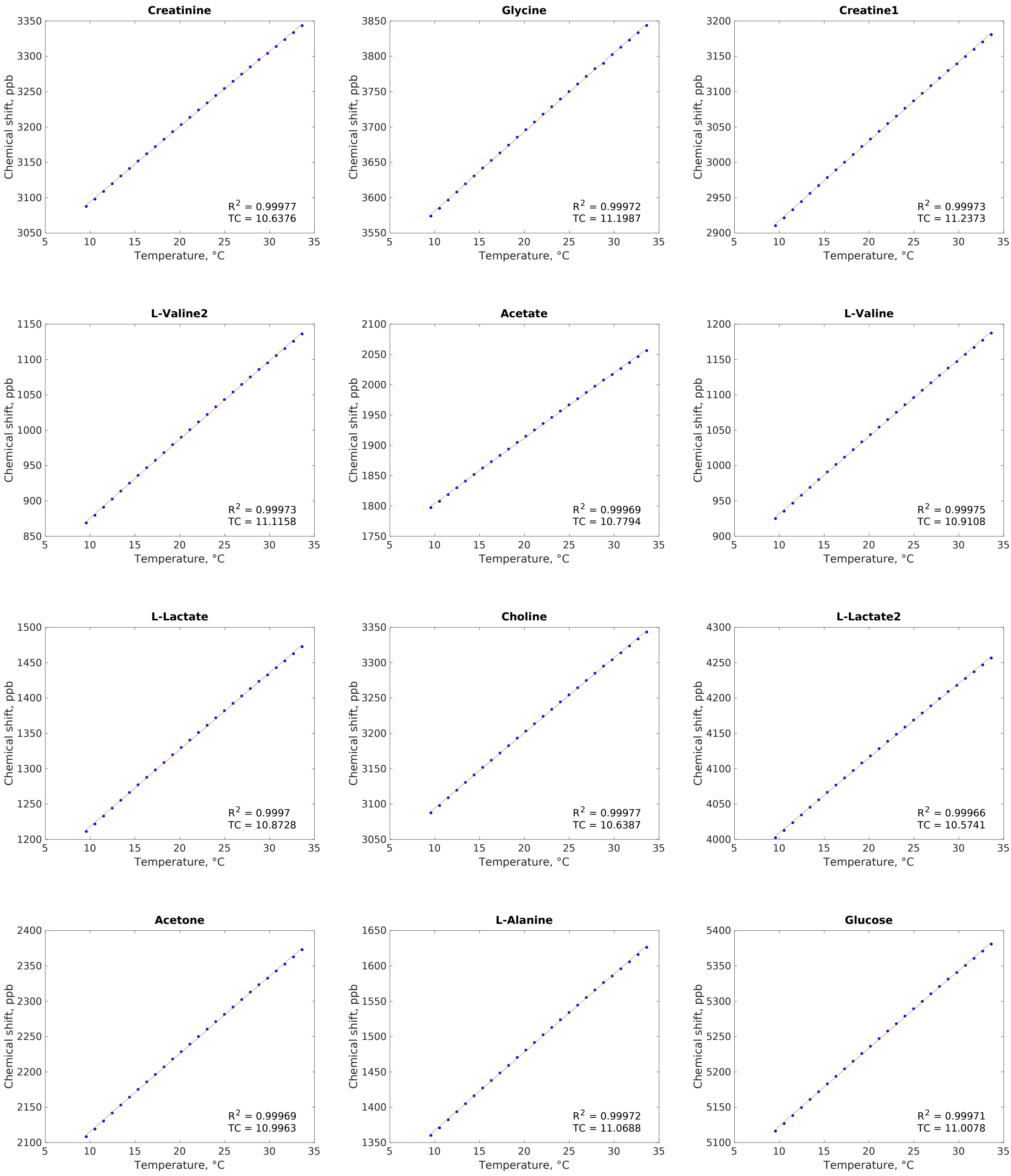

Creatinine
R^2 = 0.99977
TC = 10.6376
Glycine
R^2 = 0.99972
TC = 11.1987
Creatine1
R^2 = 0.99973
TC = 11.2373
L-Valine2
R^2 = 0.99973
TC = 11.1158
Acetate
R^2 = 0.99969
TC = 10.7794
L-Valine
R^2 = 0.99975
TC = 10.9108
L-Lactate
R^2 = 0.9997
TC = 10.8728
Choline
R^2 = 0.99977
TC = 10.6387
L-Lactate2
R^2 = 0.99966
TC = 10.5741
Acetone
R^2 = 0.99969
TC = 10.9963
L-Alanine
R^2 = 0.99972
TC = 11.0688
Glucose
R^2 = 0.99971
TC = 11.0078
Chemical shift, ppb
Temperature, °C

Chemical shift dependence[1], ppb, on temperature, °C, for NIST1950 plasma sample (experiment lasting 1 h). Temperature coefficients (TCs) as a slope of the linear function. For all diagrams, the coefficient of determination R^2 is shown.

1 Raw data, before referencing to Lactate peak

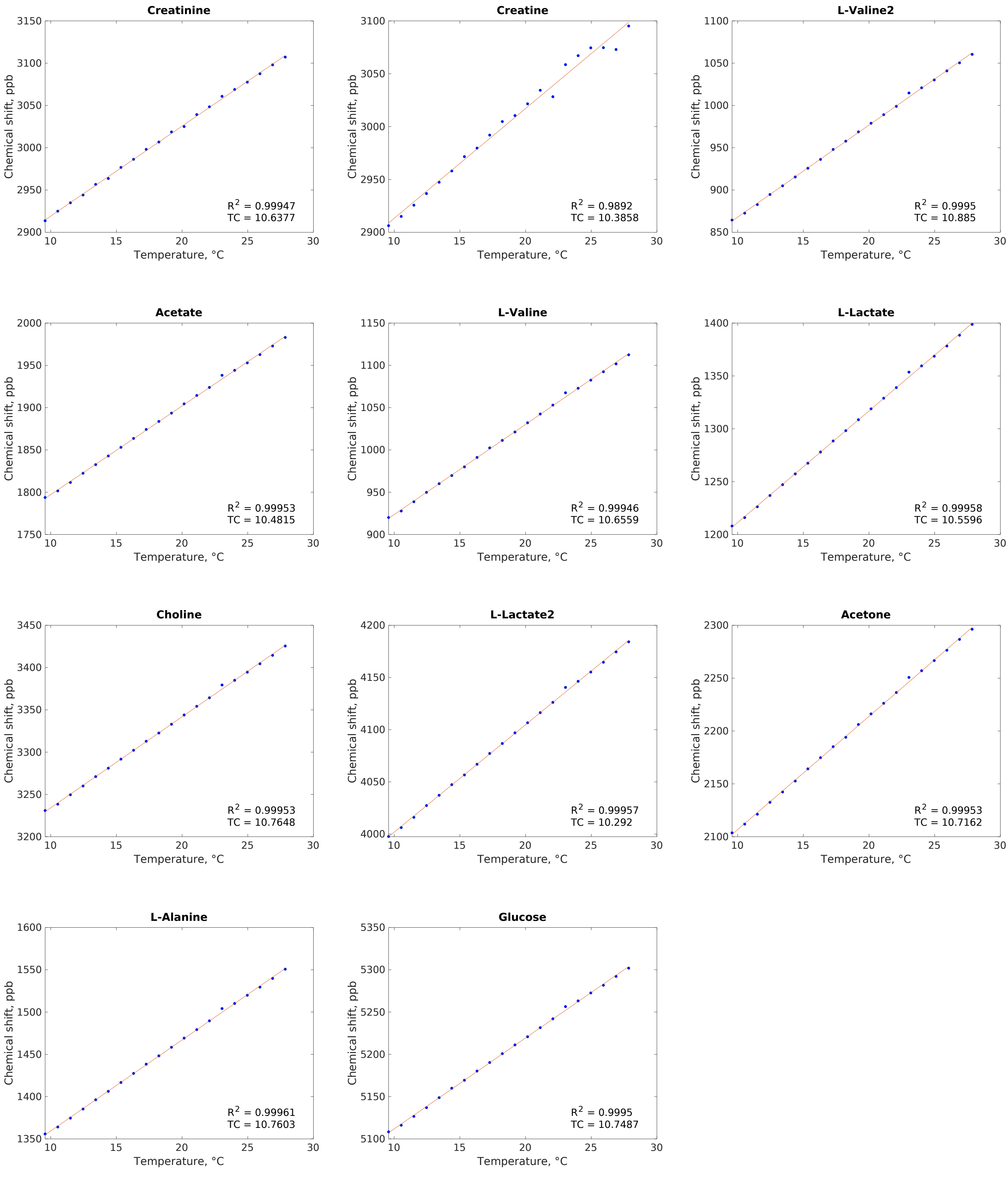
Creatinine
R^2 = 0.99947
TC = 10.6377
Creatine
R^2 = 0.9892
TC = 10.3858
L-Valine2
R^2 = 0.9995
TC = 10.885
Acetate
R^2 = 0.99953
TC = 10.4815
L-Valine
R^2 = 0.99946
TC = 10.6559
L-Lactate
R^2 = 0.99958
TC = 10.5596
Choline
R^2 = 0.99953
TC = 10.7648
L-Lactate2
R^2 = 0.99957
TC = 10.292
Acetone
R^2 = 0.99953
TC = 10.7162
L-Alanine
R^2 = 0.99961
TC = 10.7603
Glucose
R^2 = 0.9995
TC = 10.7487
Chemical shift, ppb
Temperature, °C

Chemical shift dependence[1], ppb, on temperature, °C, for metabolites from the artificial mixture 2. Temperature coefficients (TCs) as a slope of the linear function. For all diagrams, the coefficient of determination R^2 is shown.

1 Raw data, before referencing to Lactate peak

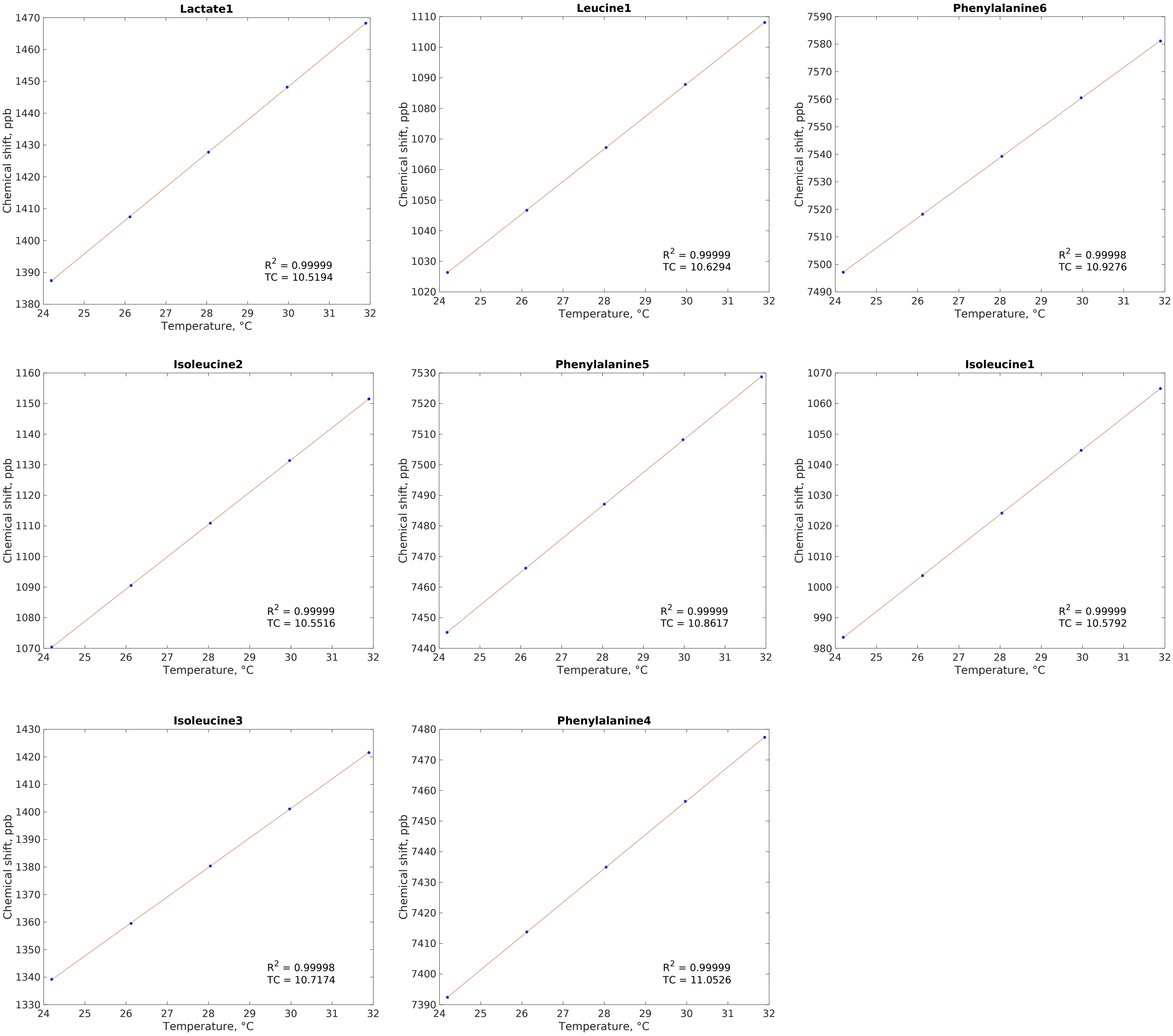
Lactate1
Chemical shift, ppb
Temperature, °C
R2 = 0.99999
TC = 10.5194
Leucine1
R2 = 0.99999
TC = 10.6294
Phenylalanine6
R2 = 0.99998
TC = 10.9276
Isoleucine2
R2 = 0.99999
TC = 10.5516
Phenylalanine5
R2 = 0.99999
TC = 10.8617
Isoleucine1
R2 = 0.99999
TC = 10.5792
Isoleucine3
R2 = 0.99998
TC = 10.7174
Phenylalanine4
R2 = 0.99999
TC = 11.0526

Chemical shift dependence[1], ppb, on temperature, °C, for metabolites from artificial mixture 3. Temperature coefficients (TCs) as a slope of the linear function. For all diagrams, the coefficient of determination R^2 is shown.

1 Raw data, before referencing to Lactate peak

S3HB - Sodium 3-hydroxybutyrate

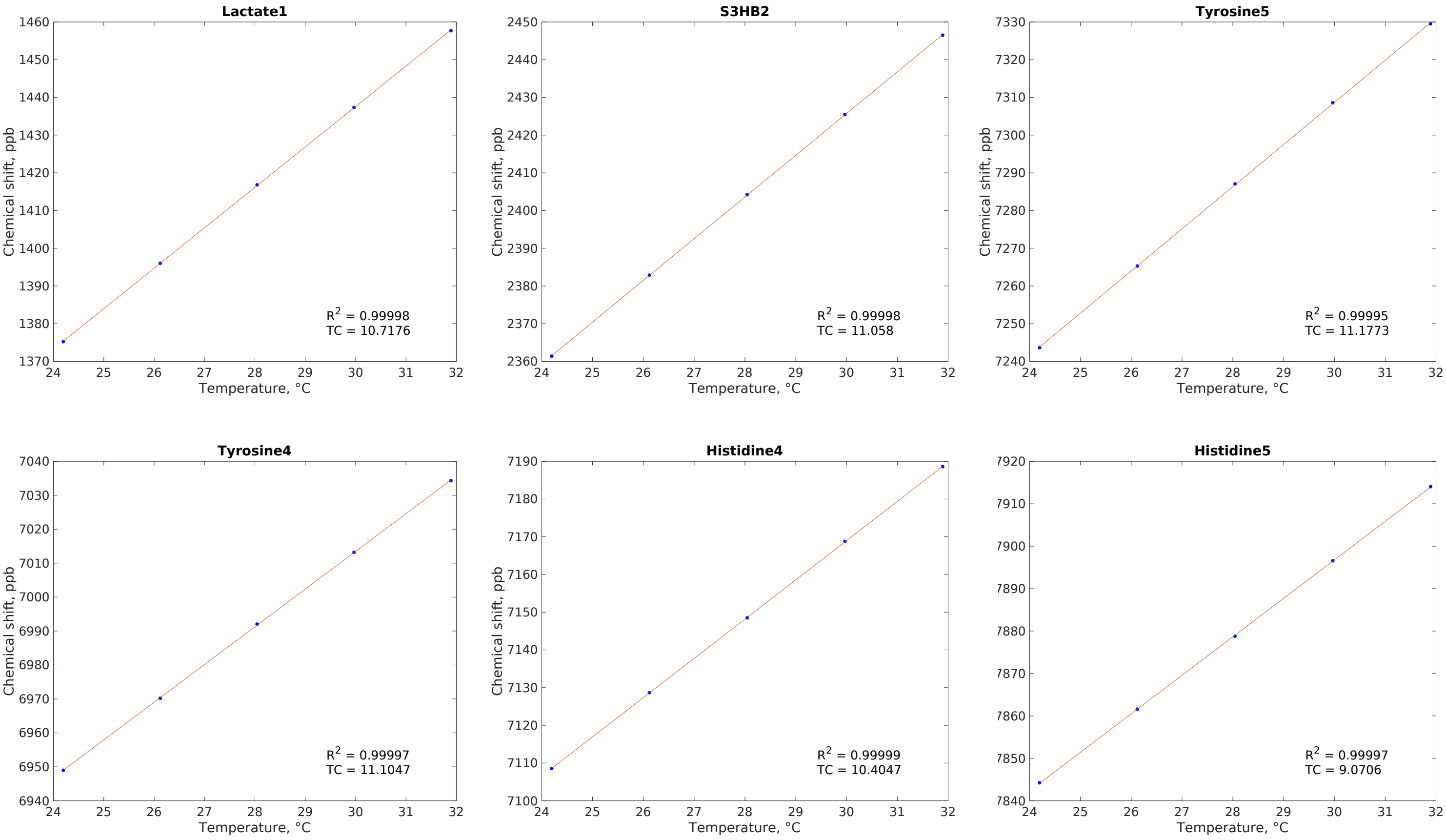
Lactate1
Chemical shift, ppb
Temperature, °C
R2 = 0.99998
TC = 10.7176
S3HB2
Chemical shift, ppb
Temperature, °C
R2 = 0.99998
TC = 11.058
Tyrosine5
Chemical shift, ppb
Temperature, °C
R2 = 0.99995
TC = 11.1773
Tyrosine4
Chemical shift, ppb
Temperature, °C
R2 = 0.99997
TC = 11.1047
Histidine4
Chemical shift, ppb
Temperature, °C
R2 = 0.99999
TC = 10.4047
Histidine5
Temperature, °C
R2 = 0.99997
TC = 9.0706

		Temperature coefficients (TC), ppb/K after referencing to Lactate peak				
	Chem. shift, ppm	Art. mixture	NIST1950 (21 h)	NIST1950 (1 h)	Mouse serum	NIST1950 (1 h) after Radon proc.
Alanine	1.51	10.93	10.93	10.95	10.94	10.90
Lactate*	1.36	10.75	10.75	10.75	10.75	10.75
Lactate	4.14	10.44	10.48	10.42	10.45	10.46
Acetate	1.95	10.64	10.67	10.63	10.65	10.66
Acetone	2.26	10.85	10.90	10.83	10.87	10.94
Choline	3.23	10.53	10.95	10.57	10.51	10.60
Glucose	5.26	10.88	10.94	10.88	10.88	10.85
Creatinine	3.07	10.87	10.82	10.77	10.51	
Creatine	3.065	11.19	10.57	11.15	11.11	11.31
Valine	1.08	10.76	10.84	10.79	10.78	10.87
Valine	1.02	10.96	11.07	10.97	10.99	11.01
Leucine	1.03	10.85	10.84		10.80	
Isoleucine	0.99	10.81	10.76		10.79	
Isoleucine	1.07	10.79	10.73		10.76	
Isoleucine	1.31	10.93	10.82		10.90	
Phenylalanine	7.39	11.27	11.30		11.47	
Phenylalanine	7.43	11.04	11.90		10.86	
Phenylalanine	7.49	11.14	11.12		11.07	
Tyrosine	6.94	11.11	11.16		11.24	
Tyrosine	7.24	11.17	11.22		11.33	
Histidine	7.11	10.45	10.58		10.61	
Histidine	7.84	9.36	9.57		9.61	
S3HB**	2.35	11.03	11.01		11.10	

* Treated as a TC reference
** Sodium 3-hydroxybutyrate
